# Safeguarding in practice: anticipating, minimising and mitigating risk in teenage pregnancy research in urban informal settlements in Nairobi, Kenya

**DOI:** 10.1136/bmjgh-2023-013519

**Published:** 2024-02-29

**Authors:** Lilian Otiso, Rosie Steege, Inviolata Njoroge, Linet Okoth, Phil Tubb, Elizabeth Nyothach, Penelope A Phillips-Howard, Sally Theobald, Miriam Taegtmeyer

**Affiliations:** 1 LVCT Health, Nairobi, Kenya; 2 Department of International Public Health, Liverpool School of Tropical Medicine, Liverpool, UK; 3 Department of Clinical Sciences, Liverpool School of Tropical Medicine, Liverpool, UK; 4 CGHR, KEMRI, Kisumu, Kenya

**Keywords:** Kenya, Maternal health, Health services research, Health systems, Public Health

## Abstract

Safeguarding challenges in global health research include sexual abuse and exploitation, physical and psychological abuse, financial exploitation and neglect. Intersecting individual identities (such as gender and age) shape vulnerability to risk. Adolescents, who are widely included in sexual and reproductive health research, may be particularly vulnerable. Sensitive topics like teenage pregnancy may lead to multiple risks. We explored potential safeguarding risks and mitigation strategies when studying teenage pregnancies in informal urban settlements in Nairobi, Kenya. Risk mapping was initiated by the research team that had prolonged engagement with adolescent girls and teen mothers. The team mapped potential safeguarding risks for both research participants and research staff due to, and unrelated to, the research activity. Mitigation measures were agreed for each risk. The draft risk map was validated by community members and coresearchers in a workshop. During implementation, safeguarding risks emerged across the risk map areas and are presented as case studies. Risks to the girls included intimate partner violence because of a phone provided by the study; male participants faced potential disclosure of their perceived criminal activity (impregnating teenage girls); and researchers faced psychological and physical risks due to the nature of the research. These cases shed further light on safeguarding as a key priority area for research ethics and implementation. Our experience illustrates the importance of mapping safeguarding risks and strengthening safeguarding measures throughout the research lifecycle. We recommend co-developing and continuously updating a safeguarding map to enhance safety, equity and trust between the participants, community and researchers.

**Summary Box**

- Safeguarding in global health is a collective responsibility for protecting people's health, wellbeing and human rights, and enabling them to live free from abuse, exploitation or neglect.
- Safeguarding risks in research can affect participants, community members and researchers.
- Multiple intersecting vulnerabilities increase safeguarding risks in studies on teenage pregnancy in informal urban settlements.
- Researchers and programmers must put deliberate efforts in place to identify and mitigate these risks before and during any study.

## Introduction

### Safeguarding

Safeguarding is a priority in global health research and development and includes protection from sexual abuse and exploitation, physical and psychological abuse, financial exploitation, bullying and neglect.^1^ We define safeguarding as measures to prevent and protect people’s (participants, communities and research staff) health, well-being and rights, including those who may be vulnerable and at risk of abuse due to the actions (or inactions) of another person(s) and or organisation(s).^1 2^ There is very little safeguarding literature from low-and-middle-income countries (LMICs) and no practical examples to help people interpret the guidelines in context. Recent UK Collaborative on Development Research safeguarding guidelines and principles in international research are designed to address this gap and support researchers to commit to and think through risks that are ‘proportionate, contextually sensitive and appropriate to the scope and nature of the research and are victim/survivor centred’.^3^ Through the Accountability and Responsiveness in Informal Settlements for Equity (ARISE) Hub (http://ariseconsortium.org/), these guidelines have been adapted further, acknowledging not everyone is equally vulnerable in informal settlements.^4^ We define vulnerability as being exposed to the possibility of physical, emotional harm or neglect. We recognise that vulnerability is unequal and is shaped by multiple intersecting power relations and axes of inequity. This work describes how intersections between poverty and power and existing high rates of structural violence in urban informal settlements shape safeguarding considerations, such as intimate partner violence (IPV).^5^ Safeguarding risks also need constant re-evaluation and these guidelines were further adapted in response to COVID-19.^6^

Safeguarding is a new and emerging field not fully addressed by research ethics, which focuses on protecting research participants during data collection. We draw on principles of ethical behaviour and specifically beneficence and non-maleficence^7^ as a strategy for researchers to address emerging safeguarding risks occurring during the study where no clear guidance is available and judgement calls are required. Researchers should be guided by situational ethics meaning responses are sensitive to and/or appropriate for the research context.^8 9^ There is need for practical examples and experience sharing on safeguarding vulnerabilities within LMICs and urban informal settlements, that help programmers, researchers and organisations address specific safeguarding issues.

### Kenyan context and intersecting vulnerabilities

Overall, 15% of adolescent girls aged 15–19 years have ever been pregnant in Kenya^10^ with up to a 21% rate of teenage pregnancy in the lowest wealth quintiles compared with wealthier ones at 8%.^10 11^ Adolescent girls in urban informal settlements face multiple intersecting vulnerabilities such as poverty, low levels of education and harmful gender norms. These result in limited autonomy, low knowledge of, and access to, contraceptives, increasing the likelihood of unintended and unwanted pregnancies.^12 13^ Kenya saw a rise in teenage pregnancy linked to COVID-19 with increasing poverty, lockdowns and school closures. School girls in Western Kenya experiencing lockdown had twice the risk of falling pregnant compared with the pre-COVID-19 period.^14^ Teenage pregnancy in Kenya is considered shameful and can result in stigma against the girl and/or family, exclusion or expulsion from school and violence from partners or parents.^15–18^ Many teenage pregnancies in Kenya stem from consensual boyfriend–girlfriend relationships^19^ but patriarchal norms mean teenage fathers may not experience the same increase in vulnerability. Some deny responsibilities, due to financial or legal constraints. Teenage fathers in Kenyan communities are also vulnerable: they may be victimised and/or criminalised for impregnating girls<18 years as per Sexual Offences Act 2006.^20^ There is limited data or discussion on teenage fathers in these settings with profound implications for safeguarding. These issues of physical, emotional and sexual violence faced by pregnant adolescents and, possibly, teenage fathers raise safeguarding concerns for researchers, programmers and organisations.

We aim to share learnings and experiences to fill the gap in literature on adolescent health, sexual health and safeguarding from LMICs and specifically urban informal settlements. In this paper, we explore how different axes of inequity shape vulnerability to safeguarding risks and present a practical approach to help programmes and researchers to address safeguarding issues for vulnerable adolescents in informal settlements.

## What we did

This study is nested within the ARISE consortium.^21^ ARISE uses co-production approaches to enhance accountability and improve the health and well-being of marginalised populations living in informal urban settlements in LMICs. We conducted an exploratory study within ARISE from 2021 to 2023 to understand the experiences of pregnant adolescents and their partners within two urban informal settlements in Nairobi—Viwandani and Korogocho—which are known to have high rates of poverty and teenage pregnancy.^11 12^

The study used a community-based participatory approach with qualitative data collection. We worked with co-researchers (young women with a history of teenage pregnancy) identified from the community, who assisted with mobilisation and follow-up of the participants. We followed up 16 purposively recruited pregnant adolescents longitudinally and carried out focus group discussions and in-depth interviews with them, their male partners, parents/guardians and community members. We explored experiences, perceptions, quality of services and accountability mechanisms.

All participants signed informed consent forms prior to participating in the study. The participant information sheet (PIS) included information about the study, potential risks to them, potential benefits to them, voluntariness of the study (freedom to choose whether to participate or not), assurance of confidentiality and their right to withdraw at any time. The participants were informed that they would be provided with a refreshment and reimbursement for their travel and time while attending the sessions amounting to Kes500 ($3.12 at current rates) in recognition that engaging with the study detracts from other income generating opportunities. After reading the PIS, adolescents less than 18 years signed their assent to take part in the study, after their parents or guardians signed the consent form on their behalf. Those 18 years and above signed their own consent forms.

The research team co-developed a safeguarding risk map (online supplemental material) based on the risk map used in the ARISE Hub study during protocol development and refined it prior to qualitative interviews. The map assessed safeguarding risks in three categories: (1) potential risks to participants, (2) potential risks to researchers and (3) safeguarding issues unrelated to research activity. The risk map was reviewed and validated during a community workshop with co-researchers, community health promoters and pregnant adolescent participants and was adopted for use in the study. Risks were then documented, discussed and reviewed reflexively during data collection and community meetings. The risks were discussed as they arose with the principal investigator (PI) and research team to agree on ways forward that minimised risks and negative outcomes to participants and researchers. Despite these measures, safeguarding risks emerged as incidents during the study or as findings during data collection.

10.1136/bmjgh-2023-013519.supp1 Supplementary data

A range of cases were discussed and documented during reflexivity meetings by the research teams following observation or interview notes at community level. We selected three cases from among these to represent typical issues and to reflect a participant risk, a researcher risk and a risk unrelated to the study as per the risk map in the paragraph above. In each case, we triangulated our findings across the experiences of participants and/or research assistants, observations and interviews and focus group discussions and existing literature to add depth to the analysis of emerging themes and priorities.

## Case study 1: how participation in a study can expose a pregnant adolescent to risk

Magdalene (pseudonym) is a 21-year-old pregnant woman. She had been enrolled as a participant in a photovoice study in March 2022 which aimed at capturing the experiences of pregnant adolescents, having falsified her age. Smartphones (approximate value US$100 similar to smartphones used in the community) were provided to participants to photograph features of their daily lives during their pregnancy and post delivery. Magdalene lived with her partner, a casual labourer. She dropped out of school at the age of 14 years. At enrolment, Magdalene was not explicitly asked whether her partner was abusive, however, she had been informed she should not participate if she deemed participation would put her at risk of any violence and consented to take part on this basis.

When the study started, the research team noticed Magdalene struggling to pay attention during meetings. On enquiry, they discovered she had been experiencing IPV. Once she came to the study meeting with bruises on her face. She had not been able to take photos as her partner had taken her phone. This was discussed as a safeguarding issue by the PI, researchers and co-researchers–it was agreed since the phone was posing a risk to Magdalene, it should be withdrawn but she could still participate in the study as an informant. When this was discussed with Magdalene, she reassured the phone would be safe with her mother, and she would collect it when needed. Unfortunately, a short while later, the partner found her taking photos in the community, insulted her and took the phone. He then left her and moved away to live with another woman.

This incident was reported to the researchers who went to collect the phone, but she reported it was unsafe to take the phone from the man, so he kept possession of the phone. Magdalene was unable to continue and withdrew from the study. We followed up with Magdalene in person and via phone repeatedly to encourage her to report the violence to the relevant authorities and get the required support in the facility, to which she declined.

To note, this was not the only case of IPV among study participants in the study site. As the research team and participants got to know and trust each other, very high rates of IPV, as defined by WHO,^22^ emerged in the informal settlements with almost all 16 participants reporting at least one incident, with IPV normalised within the broader community context.

### Emerging issues/discussions

This case study illustrates the risks women and girls face because of being enrolled in a study and benefitting from what it offers. It aligns with the first risk of the risk map: *potential risks to participants*. It highlights an important safeguarding threat to a study on vulnerable girls or women–the ongoing risk of violence. This is critical in communities, such as urban informal settlements, with high rates of violence occurring among 38% of women.^5^ Sadly, the risk of violence for women and girls in these communities is high whether in the study or not, but study processes may exacerbate risk. An emerging question is: ‘*Who chooses if a participant should be unenrolled from a study if it emerges that she may be at risk of violence–the researchers or the participants?*’

Studies, including ours, have shown vulnerable participants would still like to be involved in research they consider beneficial to themselves even though they faced potential harm.^23^ In societies where girls’ and women’s decision-making is limited, denying autonomy in decision-making may do more harm than good. Magdalene wanted to participate despite the known risk. This may stem from her stated desire to keep the phone, the financial benefit or the privilege of being heard. A systematic review of perceptions of participants in trauma-focused research found it rewarding and beneficial to participate, with only a minority of participants experiencing distress.^24^

*Should risk of violence be an inclusion/exclusion criterion in a study like this?* Excluding participants who are at risk would be considered gatekeeping and has potential to deny participants potential benefits including researching and addressing risk.^25^ Further, it limits opportunity to understand and address risks faced by vulnerable participants. Close follow-up and risk mitigation is required as part of safeguarding.

*Did the phone add an additional risk to the participant? What would be the alternative?* Providing phones or other devices and incentives has been shown to have positive and negative outcomes. A systematic review by Jennings and Gagliardi (2013) found that giving smartphones to study participants posed risks to women and led to appropriation of phones from male partners in patriarchal communities,^26^ as occurred in Magdalene’s case. On the other hand, access to smartphones through interventions can increase girls’ and women’s decision-making, social status in the community and with their partners and access to health resources.^27^

## Case study 2: potential risks to male partners during research on pregnant adolescents

The Kenyan Sexual Offences Act 2006 deems ‘impregnating girls under 18 years of age’ as illegal, regardless of the age of the male partner.^20^ If charged, men can be imprisoned for 15–20 years, though it is hotly debated as to whether the Act should apply to males under 18 years.^28^ Many male partners in our study were teenagers and we used our initial safeguarding map to identify the risks they may face:

1. Victimised and/or criminalised for impregnating girls<18 years.
2. Compelled by the family of the girls to take care of the girl/pregnancy.
3. Face stigma and violence from the community for impregnating underage girls.

We based these assumptions on an earlier study we conducted on teenage pregnancies in a rural Kenyan setting where the Chief reported that his duty was to investigate and arrest the ‘culprit’ when a teenage pregnancy is reported. He stated that most people do not report the teenage pregnancy, fearing reprisal. This led to young men not owning up, disappearing and/or being protected by the teenage mother’s family.

In the current study in informal settlements, the adolescent boys and young men expressed fear of being arrested. Some stated that the threat of being arrested alongside inability to financially provide for the girl drove them to abandon the teenage mothers. The parents of teenage mothers felt some of the boys abandoned teenage mothers for fear of being arrested, as shown in the following interview with the mother of a pregnant teenager:

Interviewer: They see like they will be jailed?

Respondent: Yes, that is what brings about difficulty to the boys.

Interviewer: According to the law?

Respondent: Sometimes they want to help the girl but they see if I go to help her, the mother will get me jailed. If the mother knows that I have made the daughter pregnant, I will not make it.

Interviewer: So, they are afraid of the law?

Respondent: Yes, they are afraid of the law…On my part when I knew that she was pregnant two months I told her to show me who made her pregnant. When I got there, I told him you walked with my daughter and now she is pregnant. That was the last day that I saw him. He moved out and went upcountry. Up to now I have never seen him again.

Notably, neither a case of arrest of any male partner was reported, nor were the cases of stigma or violence from the community against the males. However, the fear of arrest ended up harming those girls who were abandoned by their partners.

### Emerging issues/discussions

The emerging safeguarding question in this case is: *Should the study include what is a risk to both the participants (the males) and the researchers at the onset? What is the duty of researchers if they find that a participant has committed a crime according to the country’s laws? Is it right for a study to enrol or seek out a participant they know to have broken the law?* The threat of arrests may make the males feel vulnerable and unable/unwilling to participate in research studies. However, just like with the female participants, failure to research this component would deny the opportunity to gain useful information beneficial for both teenage mothers and their male partners.^25^

This case study aligns with the first and third risks of the risk map: *potential risks to participants* and *safeguarding issues unrelated to research activity* (as the male partners face risks whether we conduct the research or not).

Researchers face an ethical dilemma—to report the ‘criminal’ or not. As seen from the ongoing debate in the country, the issue of arresting the male partners under 18 years is contentious.^28^ Consensual sex between minors is not universally accepted as a crime and is therefore more a moral than criminal issue. As reported in other studies, researchers have a duty to protect the confidentiality of participants including those perceived to have committed a crime.^29^ Engagement with the community at the onset would be critical in such cases to understand their perception of the legal dilemma. For example, if following community consultation, it is not widely considered to be a crime, the researchers would be safeguarded by respecting/following the practices of the community and ethical behaviour principles.^7^ However, as stated in the participant information sheet, if the researchers identified that a serious crime had been committed, for example, rape, then they would be compelled to report it to the authorities^30^

## Case study 3: how research staff can be impacted by emotional and physical risks during a study

Our safeguarding map considered potential risks to researchers as both physical risks and psychological burn-out. Both were observed during the study. This case aligns with the second risk of the risk map: *potential risks to researchers*.

Psychological risks reported by researchers during reflexivity sessions included feeling overwhelmed by the participants’ daily struggle with poverty and suffering. Research assistants found themselves contributing their own money to provide food for the participants. In many cases, researchers would receive requests for financial support, for example, calls at night to ask for money for food. Sometimes, there was resentment from participants if the research assistants declined to send them money. The researchers ended up feeling responsible for the welfare of the participants, whom they had built a relationship with. The researchers felt their trust was abused when participants lied about their age and pregnancy status to get into the study.

The project provided counselling support for the researchers to address distress they experienced. They were able to access both group and individual counselling and reported they found counselling sessions very useful. The need for researcher counselling had not been anticipated at the beginning of the study but became critical as the reflexivity sessions were held.

Physical risks were experienced when the researchers were threatened by partners of the study participants—as illustrated in case study 1. Although none of the research assistants were physically harmed, they were placed at increased risk. In another incident, a research assistant was robbed on route to the study site: her phone was stolen and despite her screams, no one assisted her. This left her feeling vulnerable and exposed. The project was able to replace her mobile phone and provide additional counselling and she felt able to continue with the study. These examples demonstrate how the physical risks impact mental well-being.

### Emerging issues/discussions

The risks faced by researchers is an area that has not received a lot of attention by researchers and ethical review boards.^31^ The following are emerging safeguarding questions to be considered: *Are there places where studies should not be carried out due to physical and psychological risks to researchers? Is counselling enough when risks occur? What role does reflexivity play*? *What protection should be provided, and should it differ based on researcher positionality?*

With regards to physical and emotional safety and selection of sites, safety of researchers and research participants is paramount.^32^ This study was conducted in settings the research teams have been working within for a long time. The teams are known by the community and implement safety procedures, for example, physical escorts, and not conducting field visits during ‘risky periods’, for example, elections. Researcher safety must be reviewed frequently, and if the threat level increases, field work should be suspended. A checklist of physical safety can be prepared and used during the study.^32^ The selection of the researchers must consider their needs, for example, those who may feel threatened or uncomfortable working in informal settlements can be paired with those more comfortable or assigned other roles. Pairing and mixing of genders and provision of additional security have been used as strategies to protect Community Health Workers (CHW) safety in the course of their work.^33^

Winfield recommends training researchers on vulnerable populations for both personal and participant safety, focusing on ensuring researchers maintain personal and professional boundaries.^34^ Reflexivity sessions and counselling support are important during data collection and other emotionally taxing research and may mitigate burn-out.^9^ From this study, researchers gave each other a lot of support during debriefing and reflexivity sessions, alongside group counselling sessions they received, as they knew the situation deeply and had a shared experience.

### Lessons learned

Lessons learnt from this study are relevant for all individuals involved in programming and research on teenage pregnancy, including researchers, academics, programme implementers, health workers, members of ethics review boards and policymakers, among others. We outline key lessons below:

1. Safeguarding risks can occur throughout the study. It is important to identify risks early, ideally through co-developing a safeguarding risk map, which is regularly reviewed and updated together with the co-researchers and all team members.
2. The development and use of a risk map provides a systematic approach to ensure different types of risks are considered and addressed within the context.
3. Ethical review boards should incorporate safeguarding during the ethical review processes. Although related, safeguarding risks go beyond the typical ethics guidelines. Researchers should therefore include safeguarding in their protocols prior to ethical review.
4. Researchers should be guided by situational ethics (considering research context) and safeguarding throughout the study.^7–9^ This would enable them to be responsive and mitigate risks to participants and researchers.
5. These safeguarding and ethical principles go beyond research and can apply equally to programmers and policymakers to support best practice in programmes.
6. It is good practice to ensure that counselling and psychosocial support is in place for the whole research team when conducting research with clear safeguarding risks.

## Conclusion

The three case studies presented bring out key aspects of safeguarding when conducting research and programmes with vulnerable groups such as pregnant adolescents in informal settlements. These cases shed further light on safeguarding as a key priority area for research ethics and implementation and bring insights from urban informal contexts to the emerging literature. Safeguarding was not the focus of this study, but the emerging risks have demonstrated the need to focus on safeguarding as a priority, and the importance of safeguarding processes in identifying and responding to risks. The findings validate the risk map as a useful guide for researchers conducting research in similar settings. We encourage researchers to use this or similar tools to guide their assessment of safeguarding risks prior to and during the research to document and share experiences to inform more critical discussion on these ethical issues. Crucially, the map should be continuously updated with input from the participants, researchers and the community to enhance safety, equity and trust.

## Data Availability

No data are available. This is a practice piece that mainly drew on our experiences of collecting data rather than the data itself. Where relevant excerpts of our data are available within the manuscript, due to the sensitive nature of the research, we cannot share the transcripts as this would violate the data sharing agreement to which participants consented and as agreed by LSTM Research Ethics Committee. Data requests may be sent to lstmrec@lstmed.ac.uk.
