# Supplementary material for: Safeguarding in practice: anticipating, minimising and mitigating risk in teenage pregnancy research in urban informal settlements in Nairobi, Kenya
Source: BMJ Glob Health. 2024 Feb 29;9(2):e013519. doi: 10.1136/bmjgh-2023-013519 (PMC10910648; doi:10.1136/bmjgh-2023-013519)
Supplement: Supplementary data [file bmjgh-2023-013519supp001.pdf]

APPENDIX (Supplementary material)

Safeguarding mapping tool used in the research study

<table>
<tr><td colspan="7">SAFEGUARDING MAPPING TOOL - v 1 April 2021</td></tr>
<tr><td>LSTM Dept</td><td>DIPH</td><td>Title</td><td colspan="4">Accountability for Universal Health Coverage: Experiences of Pregnant Adolescent Girls in Urban Slums in Kenya</td></tr>
<tr><td>Sum mary</td><td colspan="6">The aim of this study is to improve the accountability and responsiveness of pregnancy care for adolescents in two urban slums in Nairobi, Kenya through participatory research. The study will involve pregnant adolescents, their male partners, their parents or guardians, community members and health providers and managers. The study will use a participatory action research design using a community based participatory research approach with co-researchers from the community leading the data collection and co-designing of interventions in participatory action research cycles.

The study will explore experiences of pregnant adolescents, their male partners and guardians in Nairobi slums in accessing and utilising health services over time, during and after delivery. It will assess the costs they incur and accountability structures that exist and if/how they involve adolescents. Data collected and experiences will inform interventions in cycles co-designed with beneficiaries.

Data will be collected through a mixture of qualitative methods (Photovoice, IDI, FGDs, KIIs and pregnant adolescent journals) and costing tools. Stakeholder workshops will be held to gain buy in, disseminate findings from the study and co-design interventions. Stakeholder workshops will ensure appropriate application of methods, and provision of advice on participants health and wellbeing.</td></tr>
<tr><td>Start Date</td><td>09/2021</td><td>End date:</td><td>09/2024</td><td>Country</td><td colspan="2">Kenya</td></tr>
<tr><td>PI</td><td>Lilian Otiso</td><td>Programme Manager</td><td colspan="2">Linet Okoth</td><td>Funder</td><td>GCRF UK</td></tr>
<tr><td colspan="2">LSTM code of conduct</td><td colspan="2">LSTM will be sponsor</td><td colspan="2">Does the programme use volunteers?</td><td>No</td></tr>
<tr><td colspan="2">Collaborating partners</td><td colspan="5"></td></tr>
<tr><td colspan="2">Safeguarding Risk Identification</td><td colspan="3">What are the risks</td><td colspan="2">How will the risks be mitigated/managed?</td></tr>
<tr><td colspan="2">1. Potential safeguarding/ protection risks for participants that may occur within/as a result of undertaking the research</td><td colspan="3">Pregnant adolescents:
(1) They may be impoverished and feel obligated to join the study for incentives
(2) Participation by girls may not be welcomed by others in family or community
(3) Pregnant adolescents may feel harassed or threatened by co-researchers or other research staff</td><td colspan="2">(1) Clarify any remunerations related to study travel, refreshments during study activities
(2) Ensure the study purpose is explained to ‘heads of household’ /family to minimise any possible conflicts plus parent-guardian consent
(3) Staff training to maintain professional boundaries
(4) Recognize- Train the Co-researchers (female peers with history of teenage pregnancy) and research assistants on safeguarding, power and positionality.</td></tr>
</table>

	(4) Pregnant adolescents may form attachments with staff, expect or hope for favours<br><br>(5) Pregnant adolescents may give away 'secrets' requiring safeguarding actions<br><br>(6) Loss of confidentiality of answers in journals<br><br>(7) Pregnant adolescents may be targeted by 'gangs' for perceived benefits<br><br>(8) Emotional/Psychological distress may arise during or because of the study<br><br>(9) Inadvertently disclosing the pregnancy to parents/guardians, partners' schools, community resulting in exclusion, physical or psychological harm<br><br>Male partners<br><br>(10) May be victimised and/or criminalised for impregnating girls <18 years as per Sexual Offences Act 2006<br><br>(11) May be compelled by the family of the girls to take care of the girl/pregnancy<br><br>(12) May face stigma and violence from the community for impregnating underage girls	possible signs of abuse, how to recognize the forms of abuse.<br>(5) Record-Teach them to take notes of what they have seen. Including: What happened? Why did it happen? When did it happen? What was said? Any other notable facts they can recall. Participant trained to keep their Journals in private place; staff trained to ensure confidentiality and anonymity maintained<br>(6) Report- Train the co researchers on the guiding policies around safeguarding including the community based complaint mechanisms. Let them share the information in time for action.<br>(7) Refer- Ensure Safeguarding Officer is contacted. If there is an immediate risk of harm to the individual, contact the Police, ring the Local Authority, and do not delay. Contact details provided for social support in local area; if needed further referral support through project stakeholders, to other services including counselling support. Engage with local gang leaders (currently involved in ARISE study) to support the study and protect the girls. Provide appropriate immediate counselling services and referrals for counselling follow-up if needed, address any safeguarding risks during reflexivity sessions<br>(8) In case criminal activity is detected in the course of data collection, report to the relevant offices including the local authority and MOH ensuring confidentiality is maintained and the risks to the participants and researchers are minimised<br><br>(9) Eligibility criteria includes disclosure to parents, partners or community<br><br>Engage the chief to ensure there is no victimisation of the male partners. Ensure there is no way to identify those taking part in the study for their opinion or for their direct experience<br><br>Only enrol those male partners who have resolved any issues with the families. If others involved they will be

		handled independently of the pregnant adolescents and their families
2. Potential safeguarding risks for staff, students, volunteers, contractors, consultants or visitors	(1) Research assistants in the field may be at risk if they work alone or work late after dusk.<br><br>(2) Covid 19 infection is a risk due to interactions with participants.<br><br>(3) Staff may be exploited by locals working in partnership.<br><br>(4) Male staff may be blamed for taking advantage of girls, sexual favours<br><br>(5) Incitement against the project by community members<br><br>(6)The Co- researchers and research assistants experiencing burn out from the experiences during interviews.	1) Ensure research only takes place during the day and strictly not after working hours.<br><br>2) Full PPE and protection measures upheld, staff trained; (see consent for details)<br><br>3) Staff to discuss with manager at early stage to prevent occurrence<br><br>4) Males trained to prevent this, no male works alone without female staff accompanying<br><br>5) Community inception meetings and continuous stakeholder meetings to update them on the study<br><br>6) Structured weekly reflexivity sessions put in place during data collection<br><br>7) Organised group counselling services availed on a monthly basis during data collection. Individual counselling provided if and when required.
3. Safeguarding issues that could arise unrelated to the research activity	(1) Girls at high levels of gender based violence in this area including forced marriage<br><br>(2) Local and national unrest can affect staff e.g. due to election violence<br><br>(3) COVID-19 transmission risk to staff and participants	(1) Contact details provided for social support in local area; if needed further referral support through project stakeholders, to other services.<br><br>(2) LVCT contingency plan for safe working; if unrest, field work stops<br><br>(3) KEMRI COVID-19 prevention in protocol.

**Additional Information**	
International and national legislation and/or guidance documents are available in relation to safeguarding, protection of children in the country you are working in?	What services are available locally as part of victim response for child / vulnerable adult protection? Please detail (e.g. child protection, GBV services, HIV services)
Guidelines for Conducting Adolescent Sexual and Reproductive Health Research in Kenya 2015.	Nearby health facilities including Korogocho health centre, Mama Lucy Kibaki hospital, FBO facilities in Viwandani).

Guidelines for conducting SRH/HIV research on Adolescents – KEMRI/NASCOP	LVCT Health DREAMS project (Korogocho) and Hope Worldwide DREAMS project (Viwandani) to offer comprehensive HIV and SRH services to adolescent girls and boys.
Violence Against Children National Plan of Response (2020-24)	Terre des Hommes – economic empowerment programs for adolescents
The National Children Policy Kenya 2010	Local children's office and social workers to link the girls to shelters and social protection interventions available.
School re-entry policy for pregnant girls	Local chief's office to address any conflicts emerging and communicate priorities to the community during community meetings.
HAART's Child and Vulnerable Adult Protection and Safeguarding Policy 2018	A directory is kept at the project sites and availed to all field staff to facilitate referrals
Sexual Offences Act 2006	

Action Plan. (What additional action (if any) do you now need to take to mitigate the risks identified)?

- Safeguarding will be discussed at the community entry meetings involving several stakeholders including the chief, local administration and MOH staff and officials to ensure community understands and contributes to safeguarding and response
- All research staff will be trained on safeguarding and protection of sexual exploitation and abuse (PSEA) and will sign against the policy to confirm they have received and understand it as well as sanctions which include dismissal from work and reporting to the police in case of violation of the law
- All study participants including the researchers will be taken through the safeguarding incidence reporting form and each given copies to for use during data collection and reporting and monitored weekly
- Safeguarding focal points who participants can approach will be identified for each study site and their contacts shared
- Debrief meeting sessions conducted with Safeguarding/PSEA as a standing agenda in all the meeting, plus bi weekly reflexivity sessions. Counselling sessions offered upon need
- The safeguarding officer in collaboration with the researchers and co researchers conduct periodic safeguarding assessments to elicit concerns to inform continuous sensitization on safeguarding and follow up on the arising cases
- Participants will be informed about safeguarding during the onboarding and consenting process
